# Machine learning to predict hemorrhage and thrombosis during extracorporeal membrane oxygenation

**DOI:** 10.1186/s13054-020-03403-6

**Published:** 2020-12-10

**Authors:** Adeel Abbasi, Yasmin Karasu, Cindy Li, Neel R. Sodha, Carsten Eickhoff, Corey E. Ventetuolo

**Affiliations:** 1 Department of Medicine, Warren Alpert Medical School of Brown University, Providence, RI USA; 2 Brown University, Providence, RI USA; 3 Department of Surgery, Warren Alpert Medical School of Brown University, Providence, RI USA; 4 Brown Center for Biomedical Informatics, Brown University, Providence, RI USA; 5 Department of Computer Science, Brown University, Providence, RI USA; 6 Department of Health Services, Policy and Practice, Brown School of Public Health, Providence, RI USA

Hemorrhage and thrombosis are major causes of morbidity and mortality during extracorporeal membrane oxygenation (ECMO). Even in a controlled setting, bleeding occurs frequently—almost half (46%) of the patients randomized to ECMO in the EOLIA trial had hemorrhage requiring transfusion [1]. The pathophysiology of these complications during ECMO is complex, dynamic and not fully understood [2]. This may explain why standard approaches to monitor coagulation are imperfect and studies that employ traditional biostatistical methods do not consistently identify common risk factors. We applied machine learning to an ECMO dataset to predict hemorrhage and thrombosis. Our hypothesis was that machine learning would accurately predict these events and identify novel factors not anticipated clinically or identified by traditional biostatistical methods.

We used a preexisting, manually extracted, adult ECMO dataset established to study anticoagulation practices and ECMO complications [3]. The dataset was first cleaned. Data were condensed to one row per patient. The mean and range were used to create new variables from continuous variables. Categorical variables were encoded as binary variables using one-hot encoding. Missingness was handled by first dropping variables’ missing values for all patients. Some missing data were recovered by reviewing the electronic health record. Seven variables were dropped to limit the potential of reverse causation artificially enhancing outcome prediction. Remaining variables still missing values (thromboelastography, anti-factor Xa levels) were dropped. Hemorrhage was defined as bleeding during ECMO requiring a transfusion and/or intervention, thrombosis as deep vein thrombosis, pulmonary embolism, ischemic stroke during or following ECMO, or ECMO circuitry change.

The study cohort included 44 consecutive patients supported with ECMO. The average age was 42 years; 66% were men. The most common indication for ECMO was acute respiratory distress syndrome (59%), and 66% were supported with veno-venous ECMO. There were a total of 19 hemorrhage events, most commonly cannulation site bleeding (42%), and 16 thrombotic events, most commonly deep vein thrombosis (81%).

We compared chi-square to five supervised classification and regression machine learning models: random forest, recursive feature elimination, decision trees, k-nearest neighbors and logistic regression. Leave-one-out cross-validation maximized the training cohort size, which allowed each patient to be used to train and test the models to minimize sample bias [4]. The models to predict hemorrhage performed better (accuracy of 58–80%) than the models for thrombosis (40–64%) (Fig. 1).

**Fig. 1:**
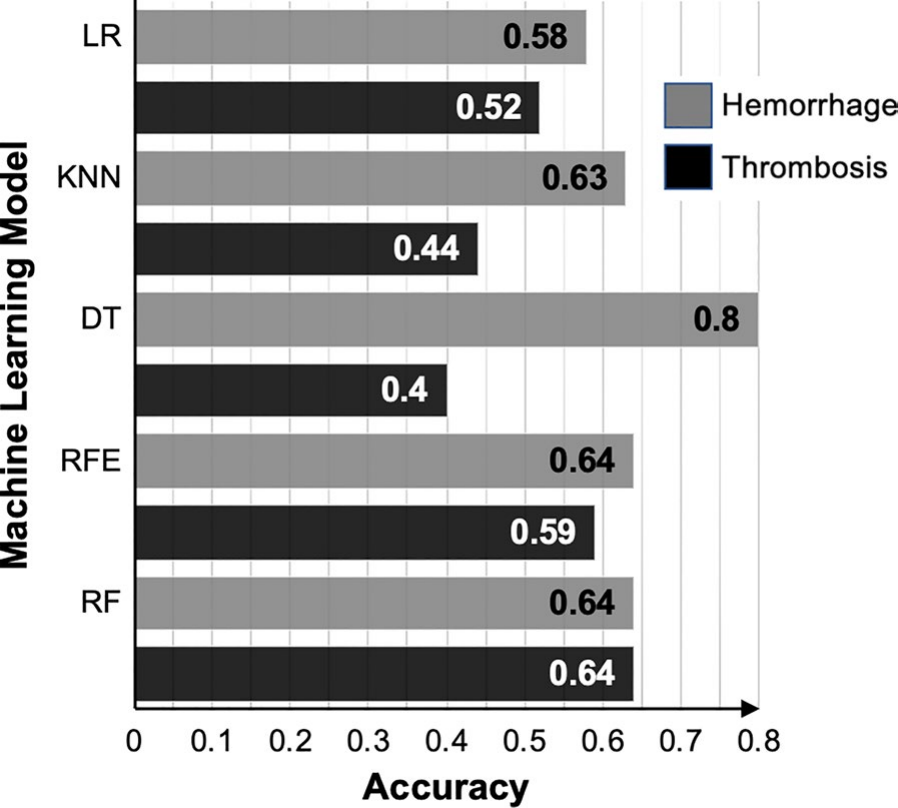
Performance of machine learning models. *DT* decision trees, *kNN* k-nearest neighbor, *LR* logistic regression, *RF* random forest, *RFE* recursive feature elimination

An ablation analysis ranked variables by importance to the model’s performance [5]. The rank lists for the random forest model differed from that of the chi-square model (Table 1). As expected, anticoagulation monitoring assays were most important in the chi-square model and the rank lists were identical for both outcomes. For the random forest model, the variables were more varied and included ECMO indications, cannulation strategies and duration. Rank lists for the random forest model differed between the two outcomes and could not be anticipated based on clinical intuition alone (e.g., race, body mass index, indication). These observations demonstrate an advantage of machine learning in its capacity to measure the correlations between *combinations* of variables and the outcome rather than correlation between the variable and outcome alone.

**Table 1:** Ten most important variables for model to predict outcomes

Random forest model*	Chi-square model
Hemorrhage	
Heparin drip rate—maximum dosage	Heparin drip rate—maximum dosage
Heparin drip rate—mean dosage	Heparin drip rate—mean dosage
PTT—lowest value	Heparin drip rate—minimum dosage
Activated clotting time—highest value	PTT—highest value
Platelet count—highest value	PTT—mean value
Race	PTT—lowest value
ECMO configuration	INR—highest value
ECMO—double-lumen cannulation	INR—mean value
Drainage cannula size	INR—lowest value
Drainage cannula site	Activated clotting time—highest value
Thrombosis	
ECMO—double-lumen cannulation	Heparin drip rate—maximum dosage
Platelet—lowest value	Heparin drip rate—mean dosage
Transfusion of cryoglobulin	Heparin drip rate—minimum dosage
Transfusion of platelets	PTT—highest value
Body mass index	PTT—mean value
Renal replacement therapy	PTT—lowest value
ECMO—duration	INR—highest value
ECMO indication—status asthmaticus	INR—mean value
ECMO indication—PH/right ventricular failure	INR—lowest value
Platelet count—mean value	Activated clotting time—highest value

*ECMO* extracorporeal membrane oxygenation, *PH* pulmonary hypertension, *PTT* partial thromboplastin time, *INR* international normalized ratio

*p > 0.05, none of the individual features significantly contributed to the model’s performance

This is the first time machine learning has been applied to predict ECMO complications. The decision tree model predicted hemorrhage with promising accuracy despite the small sample size. A larger dataset would allow the use of deep learning models to potentially improve performance and validate our current models. Similar analyses using traditional biostatistical methods are infeasible. Machine learning provides an unbiased, robust and automated approach to handle and process the volume and variety of data generated by the provision of ECMO in order to elucidate factors that contribute to ECMO complications.

## Data Availability

Please contact the corresponding author to request the data.
